# Quality measures for fully automatic CT histogram-based fat estimation on a corpse sample

**DOI:** 10.1038/s41598-022-24358-4

**Published:** 2022-11-23

**Authors:** Sebastian Schenkl, Michael Hubig, Holger Muggenthaler, Jayant Subramaniam Shanmugam, Felix Güttler, Andreas Heinrich, Ulf Teichgräber, Gita Mall

**Affiliations:** 1 Institute of Legal Medicine, Jena University Hospital, 07749 Jena, Germany; 2 Institute for Diagnostic and Interventional Radiology, Jena University Hospital, 07749 Jena, Germany

**Keywords:** Anatomy, Medical research, Mathematics and computing

## Abstract

In a previous article a new algorithm for fully automatic ‘CT histogram based Fat Estimation and quasi-Segmentation’ (CFES) was validated on synthetic data, on a special CT phantom, and tested on one corpse. Usage of said data in FE-modelling for temperature-based death time estimation is the investigation’s number one long-term goal. The article presents CFES’s results on a human corpse sample of size R = 32, evaluating three different performance measures: the τ-value, measuring the ability to differentiate fat from muscle, the anatomical fat-muscle misclassification rate D, and the weighted distance S between the empirical and the theoretical grey-scale value histogram. CFES-performance on the sample was: D = 3.6% for weight exponent α = 1, slightly higher for α ≥ 2 and much higher for α ≤ 0. Investigating τ, S and D on the sample revealed some unexpected results: While large values of τ imply small D-values, rising S implies falling D and there is a positive linear relationship between τ and S. The latter two findings seem to be counter-intuitive. Our Monte Carlo analysis detected a general umbrella type relation between τ and S, which seems to stem from a pivotal problem in fitting Normal mixture distributions.

## Introduction

In forensic temperature based death time estimation (TDE) casework rectal temperature measurement is the method of choice, the geometry and material parameters of the measurement locus’s actual surrounding are important for modelling the cooling process. Its low conductivity (see e.g.^1^ Table 6.23) makes body fat quantity and localization a potentially important issue in modelling post mortem temperature decline. The Finite Element Method (FEM) for TDE was developed to compute physics-based body core cooling. Using a CT scan to generate an FE mesh^2,3^ seems promising. One step is segmentation: matching a ‘material label’ to each of the CT scans pixels. The CT grey-scale values of body fat from muscle- and organ tissue are similar (see e.g.^2,3^), making segregating challenging. In^4^ we described an algorithm “CT histogram-based Fat Estimation and quasi-Segmentation” (CFES) for fat—muscle segmentation which was applied to only one human corpse and validated against synthetic and CT phantom data. The actual paper now presents CFES results on a sample of R = 32 CT scans of human corpses.

All three quality measures used, the τ-value (signal theory based), the anatomical misclassification rate D, and the distance measure S applied to the grey-scale value histogram, depend on CFES’s weighting exponent α (see^4^) directly as S or indirectly, as τ and D do.

## Methods

The study was reviewed and approved by the ethics committee of the University Hospital Jena. All of our experiments were performed in accordance with the relevant guidelines and regulations (see subsection ‘Ethics approval’ below).

Fat tissue (***fat***, abbreviated ‘F’) has an expectation value of the CT attenuation equivalent below the water value (0 HU), while the value of muscle-, organ-, and connective tissues (***muscle*** or ‘M’) is slightly above 0 HU (see e.g.^5^).

We will use the assumption that to any pixel in every slice a material type M_i_ can be assigned unambiguously accepting a small amount of misclassification. The term ***FM frequency*** denotes the number of pixels of a fixed grey-scale value in a CT slice.

In statements, true for fat as well as for muscle, we write Ξ as a symbol to be substituted by the letter ‘F’ for fat or by an ‘M’ for muscle. For any finite set D the symbol #D means the number of elements in D. For any function f and any set B in the preimage of f, the term f(B) stands for the set of images f(b) of elements b in B. The asterisk ‘*’ attached to a quantity-symbol means the estimated value of the quantity, whereas an attached cross ‘+’ means the true value of the indexed quantity. Generally symbols without ‘*’ or ‘+’ index are random variables. If dependence of a random variable on the real number parameter α is emphasized, the variable is indexed by ‘_α_’. For any quantity U—which is interpreted as a random variable—and any set V in the image of U the symbol μ_V_(U) stands for the mean of the quantity U, where the variation of U is bounded to the set V of possible U-values. For our list of symbols we assume for conciseness, that the slice contains fat and muscle pixel only. This is not the case in reality which is considered by constraining to the image parts where F and M are the only components. We use the abbreviations LS for Least Squares and WLS for Weighted Least Squares to specify estimation procedures.

For conciseness we will omit arguments or indices of quantity symbols if the omitted parts are unambiguously clear from the context.

We suppose the random variables y_q_ for q = 1, … , Q in a slice Y to be stochastically independent.

The problem of fat-muscle quasi-segmentation, called ***FM quasi-segmentation*** in the following, makes it necessary to distinguish the partial histograms X_F_ and X_M_ of the grey-scale value histogram X(Y) of a slice Y. As X_F_ and X_M_ are connected to the grey-scale value distributions f_F_ and f_M_ which are amalgamated to f = z∙f_F_ + (1 − z)∙f_M_ we chose the approach to firstly estimate z, f_F_ and f_M_ from the data. This enables us to directly compute the pixel numbers Q_F_ and Q_M_ solving the additional problem of ***FM quantification***. Moreover the FM quasi-segmentation problem can be solved via definition of a threshold t_FM_ in the grey-scale value histogram segregating X_F_ as effective as possible from X_M_.

For all computations we used the 8-bit CT image version resulting in N = 256 possible grey-scale values. Though this is a reduced grey-scale value resolution compared to the original 12-bit version, it yielded a better filling of the histograms compartments. Since the images were of format 512 × 512 pixels, we had Q = 262,144 pixels bearing grey-scale values for our histograms X(Y).

## The signal-to-signal separation measure τ

The classical signal-to-noise ratio (SNR) (see e.g.^6^) in the field of digital image processing is usually defined as the quotient of the mean signal amplitude μ(signal) and the standard deviation of the noise σ(noise):

1 $$SNR{ := }\frac{{\mu \left( {signal} \right)}}{{\sigma \left( {noise} \right)}}$$

As the SNR was originally designed to quantify the detectability of a narrow signal peak in a broad band of noise in frequency domain, its concept has to be adapted to our detection scenario, where the separability of two distinctly narrow signals peaks has to be measured. We will define something like an analogon τ to the SNR for our FM separation problem. The measure τ will be referred to as a ‘signal-to-signal ratio’. The grey-scale value pdfs f_Ξ_(y,Θ) of fat (Ξ = F) and muscle (Ξ = M) in a CT slice are Normal distributions Φ(y,E_Ξ_,S_Ξ_) (see^6^) in good approximation, completely determined by their moments E_Ξ_, S_Ξ_. Therefore we look for a separability measure τ depending on E_Ξ_, S_Ξ_. The separability τ should become better with growing distance of the maxima E_F_ and E_M_. The τ value should be rising with falling widths S_F_ and S_M_ making the peaks in the grey value histogram sharper. Both effects lead to smaller overlap of f_F_ and f_M_. The problem shows obvious parallels to the problem addressed by Welch’s t-test^7^. Thus the ***FM separability*** τ, as applied to a CT slice Y, is defined as:

2 $$\tau \left( Y \right) = \frac{{E_{M} - E_{F} }}{{\sqrt {\frac{1}{{Q_{M} }} S_{M}^{2} + \frac{1}{{Q_{F} }} S_{F}^{2} } }}$$

Inserting the definition of the pixel numbers Q_F_ = z∙Q and Q_M_ = (1 − z)∙Q of the components F and M into (2 we yield:

3 $$\tau \left( Y \right) = \frac{{\sqrt Q \cdot \left( {E_{F} - E_{M} } \right)}}{{\sqrt {\frac{{S_{F}^{2} }}{z} + \frac{{S_{M}^{2} }}{1 - z}} }}$$

Since there is no direct access to the actual values of the variables E_F_, E_M_, S_F_, S_M_, z one has to approximate them in formula (3) by using estimators E_F_*, E_M_*, S_F_*, S_M_*, z* instead, yielding an estimator τ* for τ.

## The anatomical FM misclassification measure D

The quality of the quasi-segmentation result W*(Y) is determined by an informed observer, comparing W*(Y) to the single CT image Y. This is formalized by the function d(Y, W*(Y)), called the (***single slice FM) misclassification***:

4 $$d\left( {Y,W^{*} \left( Y \right)} \right){ := }\left\{ {\begin{array}{*{20}c} 0 \\ 1 \\ \end{array} if \begin{array}{*{20}c} {No\,anatomical\,error\,detected\,comparing\,Y\,to\,W^{*} \,\left( Y \right)} \\ {Anatomical\,error\,detected\,comparing\,Y\,to\,W^{*} \,\left( Y \right)} \\ \end{array} } \right.$$

Given a CT scan Y = {Y_1_, … , Y_L_} of an human abdomen we define the ***(FM) misclassification rate*** D(Y, W*(Y)) as the mean of the single-slice FM misclassification:

5 $$D\left( {\underline {Y} ,W^{*} \left( {\underline {Y} } \right)} \right){ := }\mu_{{\underline {Y} }} \left( {d\left( {Y,W^{*} \left( Y \right)} \right)} \right) = \frac{1}{{\# \underline {Y} }}\mathop \sum \limits_{{Y \in \underline {Y} }} d\left( {Y,W^{*} \left( Y \right)} \right) = \frac{1}{L}\mathop \sum \limits_{{Y \in \underline {Y} }} d\left( {Y,W^{*} \left( Y \right)} \right)$$

The ***(sample FM) misclassification rate*** F(Ψ,W*(Ψ)) refers to a sample Ψ = {Y_1_, … , Y_R_} of CT scans Y_r_ of different human abdomina analogously:

6 $$F\left( {\Psi ,W^{*} \left( \Psi \right)} \right)\,{ := }\,\mu_{\Psi } \left( {D\left( {\underline {Y} ,W^{*} \left( {\underline {Y} } \right)} \right)} \right) = \frac{1}{\# \Psi }\mathop \sum \limits_{{\underline {Y} \in\Psi }} D\left( {\underline {Y} ,W^{*} \left( {\underline {Y} } \right)} \right) = \frac{1}{R}\mathop \sum \limits_{{\underline {Y} \in\Psi }} D\left( {\underline {Y} ,W^{*} \left( {\underline {Y} } \right)} \right)$$

The values d(Y, W*(Y)), D(Y, W*(Y)) and F(Ψ,W*(Ψ)) depend on the CFES’ parameter value α via W*(Y) of the scans Y in Ψ. If this fact will be emphasized in the following we will append an index α to the respective symbol, as in: W*_α_(Y), d(Y, W*_α_(Y)), D(Y, W*_α_(Y)), F(U, W*_α_(U)). If we talk generally about the influence of parameter α on FM misclassification, we use the symbols d_α_, D_α_, F_α_.

## The weighted least squares distance S_α_

The ***(WLS) distance*** S_α_(X(Y), X(Θ)) of a slice Y with reference to a parameter vector Θ, which was used as target parameter for CFES’ nonlinear estimation process, can be thought of as another quality measure defined. It is computed from the empirical histogram X(Y) : = {x_0_(Y), … , x_N-1_(Y)} and the theoretical histogram X(Θ) : = {x_0_(Θ), … , x_N-1_(Θ)} as follows:

7 $$S_{\alpha } \left( {X\left( Y \right), X\left( \Theta \right)} \right){ := }\mathop \sum \limits_{{n \in G_{0} }} \left( {x_{n} \left( Y \right) - x_{n} \left( \Theta \right)} \right)^{2} \cdot x_{n} \left( Y \right)^{\alpha }$$

As a seemingly evident hypothesis we primarily assume:

(S): For any CT slice Y: A small value of the distance S_α_(X(Y), X(Θ)) between measured grey value histogram X(Y) and theoretical grey value histogram X(Θ) given an actual value of the parameter Θ, or a high value of the FM separability τ_α_(Y), should lead to a good FM quantification in terms of a correctly estimated value of the FM proportion parameter z as well as to a good FM quasi-segmentation measured via the single-slice FM misclassification d_α_(Y,W*(Y)).

The minimisation of (7) as well as the formulae for the sensitivity analysis of the WLS approach are described in more detail in the supplementary information. There we derive the equations (S1.3) for small deviations of the WLS parameter estimation, the components of the Jacobi matrix (S2.1)–(S2.13), the components of the estimators covariance matrix (S3.3) and explain our assumption (S4.3) for the covariance matrix of the input, the grey-scale value histogram.

## The sample of human body scan data

CFES was applied to CT scans of human abdomens. The abdomen’s upper and lower bounds were defined by anatomical landmarks: The central diaphragm region characterizes the upper scan limit, while the visible conjunction of the upper part of os pubis (facies symphysialis) marks the lower limit.

After excluding three of the originally 35 corpses from the sample due to missing CT data or due to artefacts deteriorating the CT scan, the sample consisted of R = 32 human bodies of 11 female and 21 male subjects. The prosecution had levied on the bodies and ordered the CT scans as well as the autopsies. For every body age (mean = 57.41 y, stddev. = 20.22 y), body weight (mean = 71.37 kg, stddev. = 16.59 kg), body height (mean = 1.70 m, stddev. = 0.08 m) and body mass index (mean = 24.43 kg/m^2^, stddev. = 4.92 kg/m^2^) were registered.

For all of the scans Y a LightSpeedVCT (GE Medical Systems) was used with a filtered back projection reconstruction, a ‘body’ filter type and with a spiral pitch factor of 0.984. Table 1 shows an overview of our scan’s CT parameter values. For our pixel-volume calculation the parameter ‘Spacing between slices’ (column 5 of Table 1), was relevant rather than the parameter ‘Slice Thickness’ (column 6 of Table1).

**Table 1:** CT parameter of the corpse sample (three of the originally 35 corpses excluded).

LFD	Tube-voltage (kVp)	Tube-current (mAs)	Convolution kernel	Spacing between slices (mm)	Slice thickness (mm)
1	120	99	Standard	3.00	3.75
2	120	120	Standard	3.00	3.75
3	120	156	Standard	3.75	3.75
5	120	400	Standard	3.00	3.75
6	120	500	Standard	0.625	0.625
7	120	500	Standard	0.625	0.625
8	120	141	Bone	3.00	0.625
9	120	500	Standard	0.625	3.75
10	120	500	Standard	0.625	0.625
11	120	460	Standard	0.625	0.625
12	120	99	Standard	0.625	0.625
13	120	400	Standard	0.625	0.625
14	120	500	Bone Plus	0.625	0.625
15	120	99	Standard	0.625	0.625
17	120	500	Standard	0.625	0.625
18	120	490	Standard	0.625	0.625
19	120	500	Standard	0.625	0.625
20	120	500	Standard	0.625	0.625
21	140	445	Bone Plus	0.625	0.625
22	120	500	Standard	0.625	0.625
23	120	500	Standard	0.625	0.625
24	120	370	Standard	0.625	0.625
26	120	500	Standard	0.625	0.625
27	120	500	Standard	0.625	0.625
28	120	500	Standard	0.625	0.625
29	140	350	Standard	0.625	0.625
30	120	425	Standard	0.625	0.625
31	120	490	Standard	0.625	0.625
32	120	500	Standard	0.625	0.625
33	140	350	Standard	0.625	0.625
34	140	350	Standard	0.625	0.625
35	140	350	Standard	0.625	0.625

## Former usage of study material

The CT-data and its CFES-evaluations were used in the thesis^27^ of Schenkl, who is the first author of the present article.

## Ethics approval

The study was reviewed and approved by the ethics committee of the University Hospital Jena. According to the ethics committee’s statement written consents of the kinship for the abdominal CT slices shown in the article were not needed since the bodies were confiscated by the local prosecution who directed the CT scans for investigations. Moreover, the representations of the slices are totally anonymized.

## Results

### Quality parameters: single analysis

**Figure 1:**
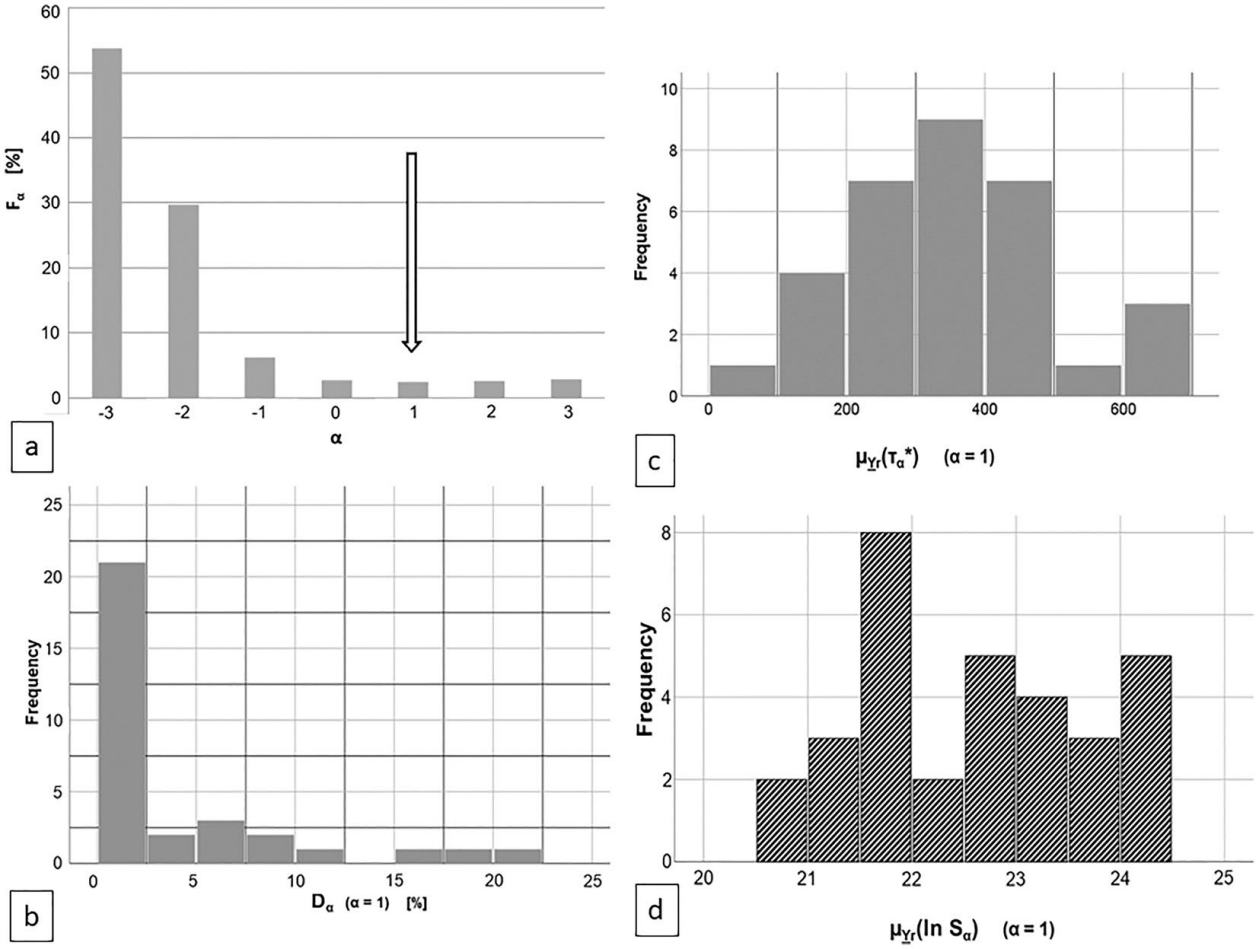
(**a**) Mean FM misclassification rate F_α_ for whole body CT sample Ψ as a function of weight exponent α. (**b**) Histogram of the FM misclassification rate D_α_ for α = 1. (**c**) Histogram of μ_Yr_(τ*_α_) with α = 1. (**d**) Histogram of μ_Yr_(ln S_α_) for α = 1.

Figure 1A presents misclassification rate F_α_(Ψ,W*(Ψ)) as a function of α. The optimum exponent α = 1 with F_α_ = 0.036 = 3.6% is marked. Figure 1b is the a misclassification rate D_α_ histogram for α = 1.

Figure 1c, d show the mean separability μ_Yr_(τ*_α_)- and the mean μ_Yr_(lnS_α_) histogram respectively, taken over all CT slices Y_r,l_ for an abdominal scan Y_r_ for each one of the R bodies.

## Quality parameters: multiple parameters analysis

**Figure 2:**
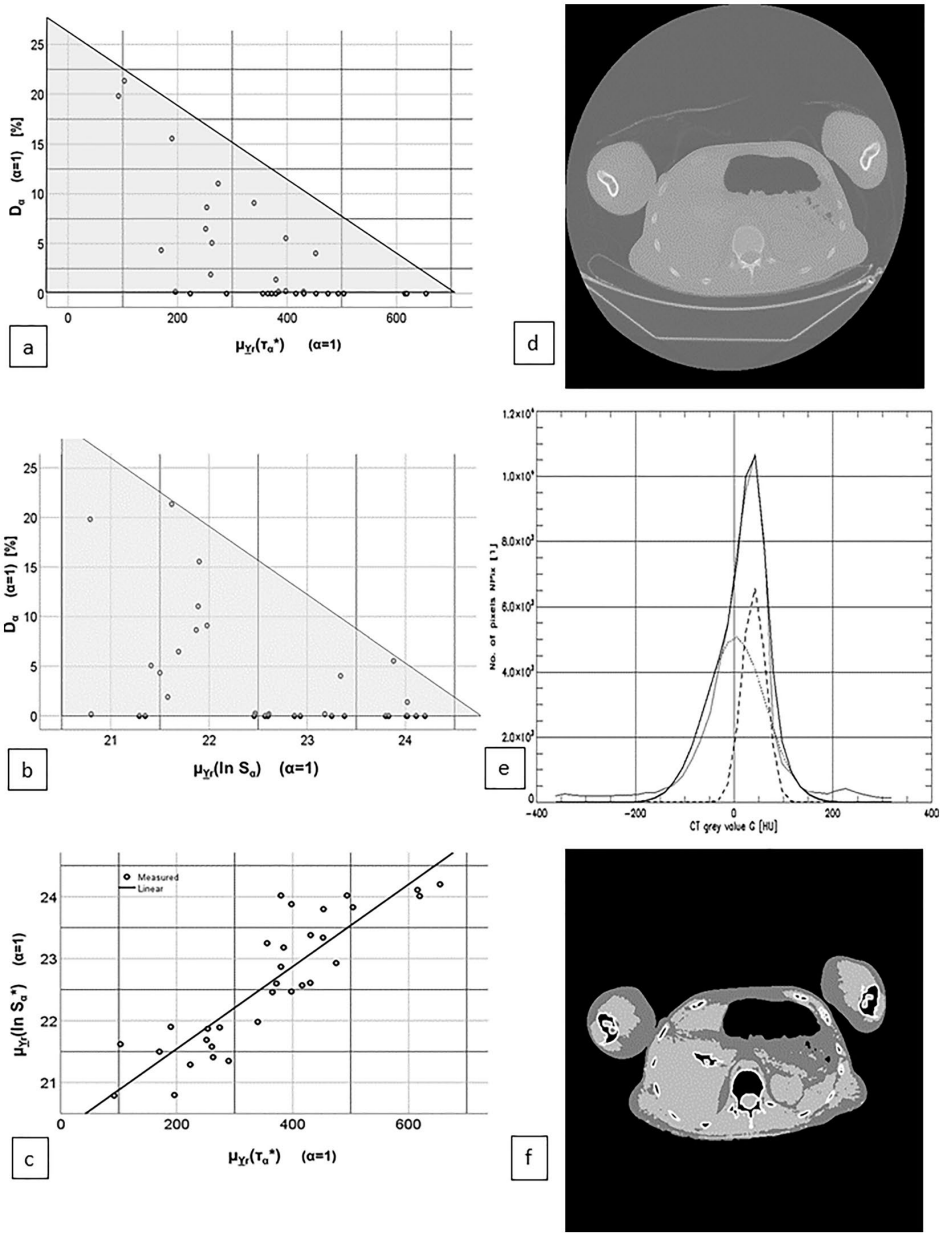
(**a**) Scatterplot: FM misclassification rate D_α_ and mean FM separability μ_Yr_(τ_α_*) of all individual scans Y_r_ in the sample Ψ for α = 1. Triangle inserted to show non-function type relation. (**b**) Scatterplot: FM misclassification rate D_α_ and mean log WLS distance μ_Yr_(ln S_α_) of all individual scans Y_r_ in sample Ψ for α = 1. (**c**) Scatterplot: mean log WLS distance μ_Yr_(ln S_α_) and mean FM separability μ_Yr_(τ*_α_) of each individual scan Y_r_ in the sample Ψ with α = 1. The drawn line represents a linear model fitted with by a least square approach. (**d**) Example slice from scan LFD_5: Original CT slice. (**e**) Example slice from scan LFD_5: Grey value histograms: measured: X (thin line), fitted: X(Θ) (fat line), partial fat: X_F_(Θ) (dotted line), partial muscle X_M_(Θ), fat vertical line: threshold t_FM_. (**f**) Example slice from scan LFD_5: Quasi segmented image (light grey: muscle, dark grey: fat, white boundary area: bones), the weight exponent was α = 1.

In Fig. 2a a scatterplot of mean separability μ_Yr_(τ_α_*) (X-axis) versus misclassification rate D_α_ (Y-axis is shown while Fig. 2b presents a scatterplot of the mean log minimal distance μ_Yr_(ln S_α_) and misclassification rate D_α_ (for both α = 1). Figure 2c is a scatter plot of mean separability μ_Yr_(τ*_α_) (X-axis) and mean log WLS distance μ_Yr_(lnS_α_) (Y-axis) with α = 1 showing a distinctive linear relation (Linear regression analysis: R^2^ = 0.772, p < 0.001, slope a = 0.007 and intercept b = 20.145).

Figure 2d–f present an example slice with μ_Yr_(τ_α_*) = 170.24/D_α_ = 4% with single-slice misclassification d_α_ = 0 but low mean separability τ_α_* = 128.35 and middle-to-high log distance ln S_α_ = 23.38 for α = 1.

As slope a > 0 contradicts the naive intuition (S) (‘high separability τ_α_ causes low distance S_α_’), we further studied the effect using simulated images.

## Quality parameters: Monte Carlo analysis of mean log WLS distance μ_**Y**r_(ln S_α_) and mean FM separability μ_**Y**r_(τ_α_*)

**Figure 3:**
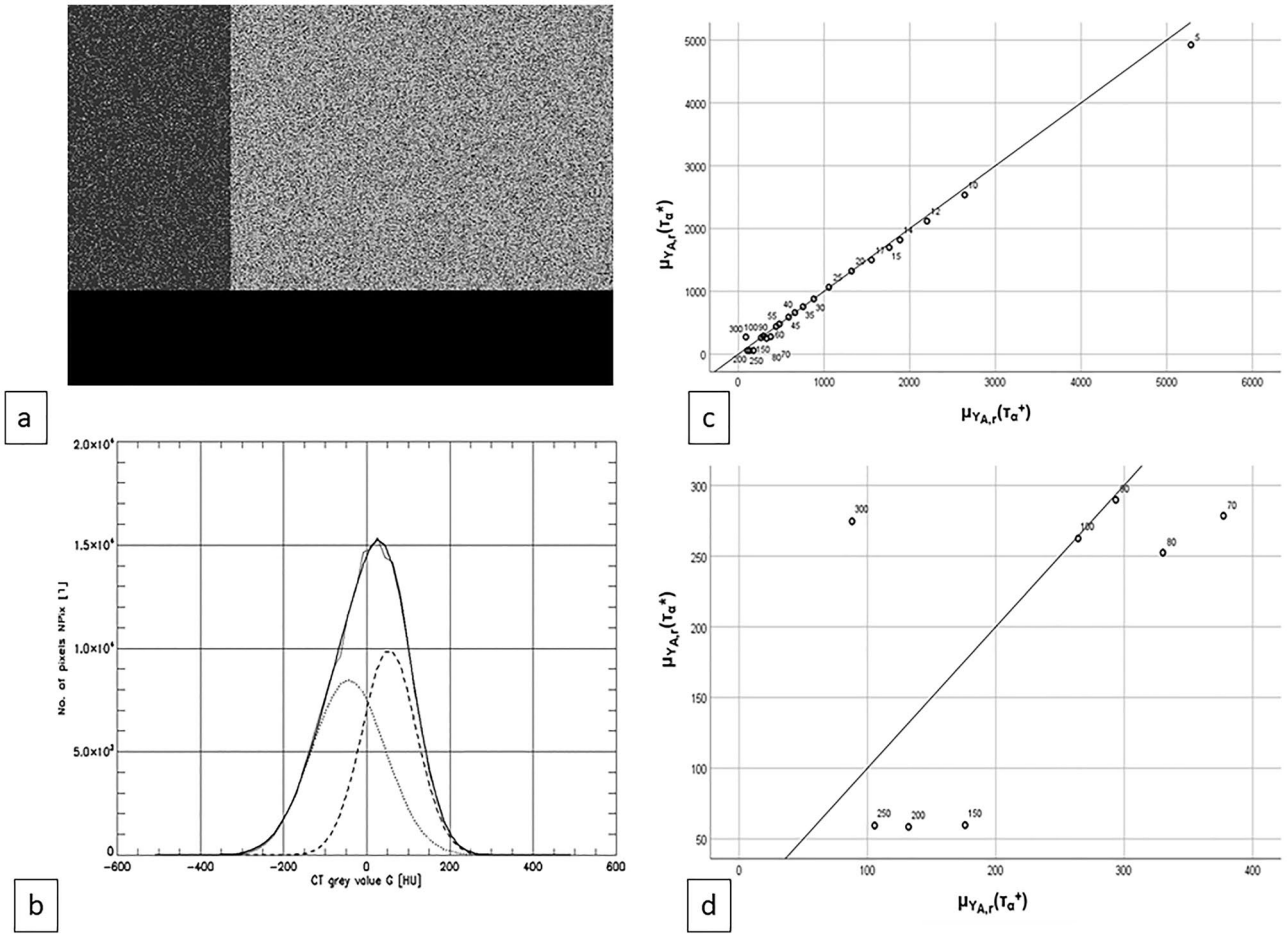
(**a**) Monte Carlo simulated CT image Y_A,r_ with components fat (dark grey), muscle (light grey), air (black). The true image parameters were: E_F_^+^ = −80 HU, E_M_^+^ = 50 HU, S_F_^+^ = S_M_^+^  = 70 HU, z^+^ = 0.3. (**b**) Grey-scale value histograms X (empirical), X(Θ) (fitted via CFES with α = 1), X_F_(Θ), X_M_(Θ) with: Thin line: measured, fat line: estimated, dotted: fat, dashed: muscle. (**c** and **d**) Scatterplots of mean true value μ_YA,r_ (τ_α_^+^) vs. mean estimated value μ_YA,r_ (τ_α_*) with z = 0.3. (**c**) The diagrams containing all of the synthetic images Y_A,r_ generated. (**d**) Diagram as in the upper right part only diagrams Y_A,r_ were considered with standard deviations S = S_F_^+^  = S_M_^+^  ≥ 70 HU. The parameter S = S_F_^+^  = S_M_^+^ labels the data-points. The weight exponent α = 1 was used in S_α_.

We used two series Y_A,1_, … ,Y_A,R_ and Y_B,1_, … ,Y_B,R_ (R = 20) of simulated images (Fig. 3a,b) with E_F_ = −80 HU, E_M_ = 50 HU and S = S_F_ = S_M_ = 10 HU, 12 HU, 14 HU, 15 HU, 17 HU, 20 HU, 25 HU, 30 HU, 35 HU, 40 HU, 45 HU, 55 HU, 60 HU, 70 HU, 80 HU, 90 HU, 100 HU, 150 HU, 200 HU, 250 HU. For series A and B respectively z was set z = 0.3 and z = 0.5. All images Y_A,r_ and Y_B,r_ were evaluated by CFES algorithm with α = 1. The true values and the estimated values of E_F_, E_M_, S_F_, S_M_, z, and the quality parameters ln S_α_, the true and the estimated value of τ_α_ were recorded.

The τ_α_^+^ vs. τ_α_*—scatterplots in Fig. 3c, d respectively represent the full sample Y_A,r_ (r = 1, … ,R) and the Y_A,r_ with τ_α_^+^-range of [0, 400] only.

**Figure 4:**
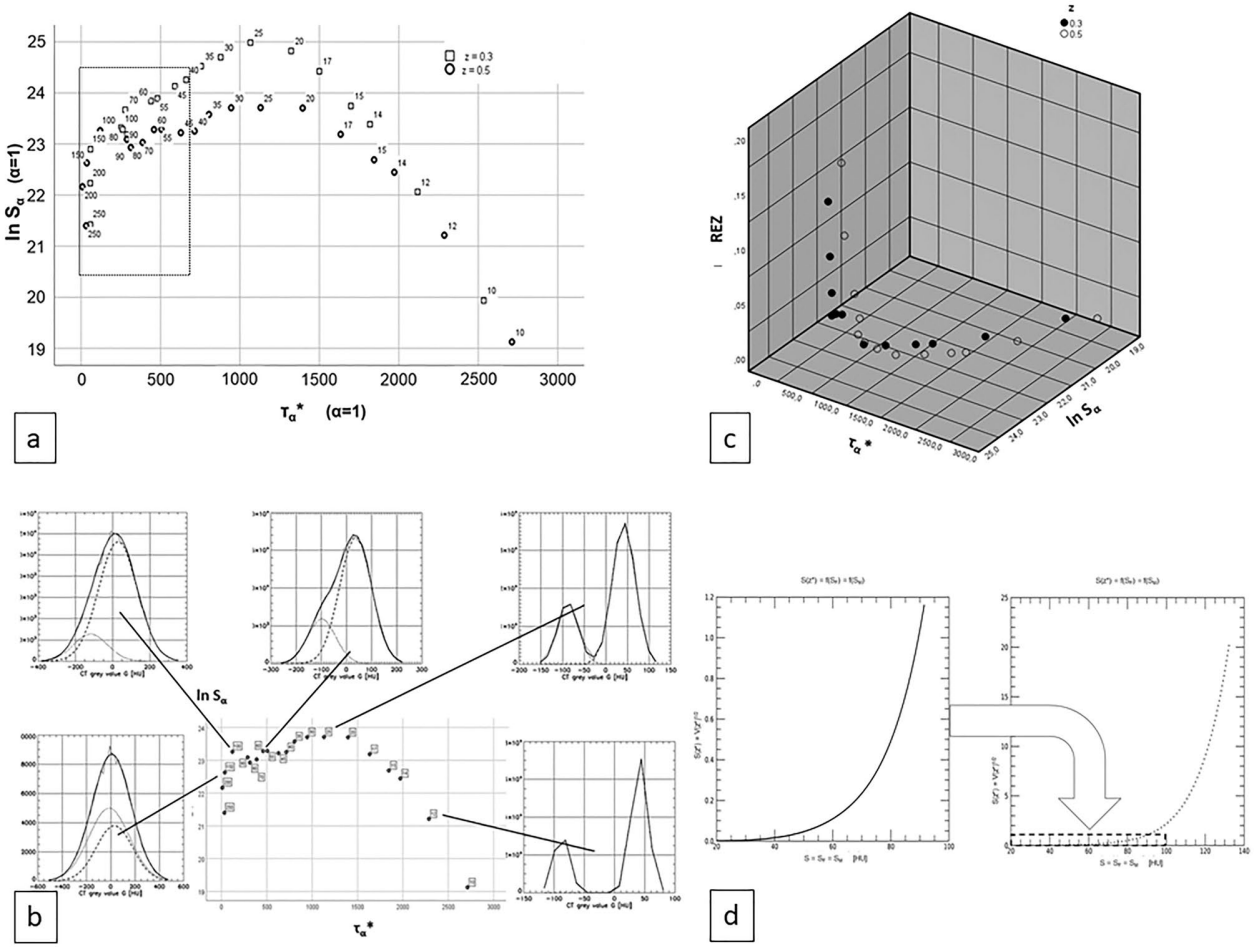
(**a**) Scatterplot for the variables FM separability τ_α_* and logarithm of weighted least squares sum ln S_α_ of the Monte Carlo images Y_A,r_ and Y_B,r_ with r = 1, … , 20 (where A: z = 0.3 and B: z = 0.5). The data-points are labelled with the number of their grey-scale value histograms true standard deviations S^+^  = S_F_^+^  = S_M_^+^. The dashed rectangle on the left side marks the τ_α_* − ln S_α_—area of the diagram in Fig. 2d. (**b**) In the middle: Scatterplot for the variables FM separability τ_α_* and logarithm of weighted least squares sum ln S_α_ of the Monte Carlo images Y_A,r_ with r = 1, … , 20. The data-dots are labelled with the value of their grey-scale value histograms true standard deviations S^+^  = S_F_^+^  = S_M_^+^. Encircling the central scatterplot five empirical and estimated grey-scale value histograms X (thin, drawn), X(Θ) (fat, drawn), X_F_(Θ) (fat, dashed), X_M_(Θ) (fat, dotted) are presented, each is connected to its corresponding data-dot in the central scatterplot by a fat drawn pointer-line. (**c**) 3-D scatterplot of the variables FM separability estimator τ_α_*, the logarithm of the weighted least squares distance ln S_α_ and relative error RE_z_ of the z-estimator for the image-series Y_A,r_ and Y_B,r_ with r = 1, … , R. Black bubbles : A: z = 0.3 void bubbles: B: z = 0.5. Images Y_A,r_ and Y_B,r_ were excluded if S ≤ 5 or S ≥ 100 as well as if RE_Z_ ≥ 0.2. The weighting exponent was set to α = 1. (**d**) Computed diagrams of the z-estimator’s standard deviation S(z*) as a function of the standard deviations S = S_F_ = S_M_ of the partial fat and muscle distributions in the scenario Y_A_ and Y_B_ with E_F_ = −80 HU, E_M_ = 50 HU, z = 0 and ranges 20 HU ≤ S ≤ 90 HU (A: left) and 20 HU ≤ S ≤ 130 HU (B: right). Note the different ranges on the ordinate. The graphs were generated with a true fat ratio value z^+^  = 0.3. However the choice of the true fat ratio value z^+^ had virtually no influence on the result when it was changed from z^+^  = 0.3 to z^+^  = 0.5. The weight exponent was chosen α = 0 which means z* is an LS-estimator rather than an WLS estimator as it is in the case α = 1.

Figure 4a is the scatterplot of ln S_α_ vs. τ_α_ over the Y_A,r_—and the Y_B,r_ sample (A: z = 0.3: fat rings, B: z = 0.5: fat rectangles). The standard deviation value S labels every data point. A dashed rectangle marks the area × [0, 700] × [20.5, 24.5] of Fig. 2c.

The center of Fig. 4b repeats Fig. 4a with associated histograms X (thin line), X_Ξ_*(Θ) (dotted: Ξ = F, dashed: Ξ = M), X*(Θ) shown for the marks S = 12 HU, 25 HU, 40 HU, 60 HU, 100 HU.

Figure 4c investigates the ‘umbrella’ in Fig. 4a, b. It shows a 3-D scatterplot (Y_A,r_ or Y_B,r_ excluded if S ≤ 5 or S ≥ 100 or if RE_Z_ ≥ 0.2.) of separability τ_α_*, logarithmic distance ln S_α_ and the relative error RE_Z_ =|z^+^  − z*|/z^+^ of z-estimation (Black filled bubbles = Y_A,r_ (z = 0.3), void bubbles = Y_B,r_ (z = 0.5)). The view is leading through the left and right front sides as well as through the top side of the cube.

Figure 4d presents the Gaussian estimation of our WLS estimation z*’s standard deviation S(z*) with α = 0, where WLS = LS and E_F_ = −80 HU; E_M_ = 50 HU, z = 0.3 (graph for z = 0.5 is nearly identical), S = S_F_ = S_M_ varied between S = 20 HU and S = 90 HU (Fig. 4d left) and with ΔE : = E_M_ − E_F_ between S = 20 HU and S = ΔE/2 = 130 HU respectively (Fig. 4d right). The curves in Fig. 4d were generated for α = 0, since this guarantees the weighting to be of no influence. The latter was preferable as for each of the values r = 1–3 the weight-matrix else would have been different for every two slices else, thus introducing noise in the evaluation.

## General results

**Figure 5:**
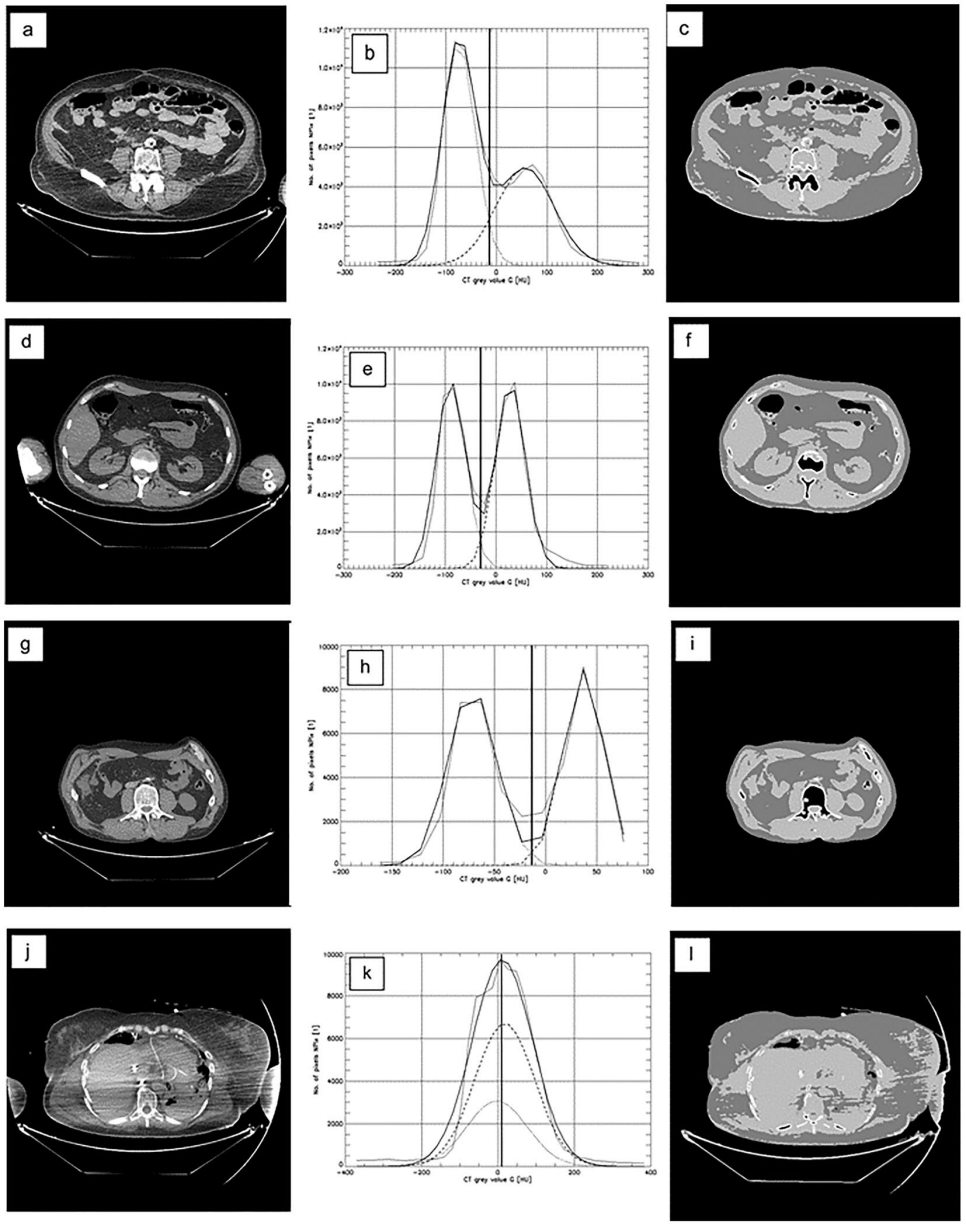
Four slices Y in (**a**), (**d**), (**g**), (**j**) from four different persons at different abdominal positions. Respective histograms with thin line = measured data X, thick line = fitted histogram X(Θ*) , partial histograms X_F_(Θ*), X_M_(Θ*) in (**b**), (**e**), (**h**), (**k**) and quasi-segmentation results W*(Y) in (**c**), (**f**), (**i**), (**l**). The parameter α was α = 1.

As examples of the CFES performance Fig. 5a,d,g,j show four slices Y, their grey-scale value histograms X(Y), X(Θ*), X_F_(Θ*), X_M_(Θ*) (Fig. 5b,e,h,k) and the respective quasi-segmentation results W*(Y) (Fig. 5c,f,i,l). Horizontal image-line 1, 2, 3, 4 corresponds to LFD 06, 22, 24, 16 (see Table 1) respectively. While the first three slices (a), (d), (g) lead to acceptable results, slice (j) was added as a poor result caused by weak contrast and artefacts. LFD 16 was excluded from our statistical evaluations.

## Discussion

### General discussion

The exponent α’s calibration in the paper^4^ yielded optimum values α = −1 for synthetic CT images and α = −1 for the real-world phantom. Application of CFES to our body CT scan sample Ψ and evaluating the misclassification rate F_α_(Ψ, W*(Ψ)) leads to Fig. 1a. This indicates α = 1 *to be the optimal parameter value with* F_α_ = 3.6% *for our body sample* though the values for α = −1, α = 2 and α = 3 lie only slightly above: The F_α_-values for α = −3, − 2, − 1 are F_α_ = 54%, 30%, 6% which is distinctively higher.

The three quality measures τ_α_, D_α_ and S_α_ are a posteriori quantities. This is evident for D_α_, and for S_α_. Only for synthetic CT images the separability τ_α_^+^ could be determined in advance. τ_α_ can only be estimated from a priori parameter estimators as described in^4^. The parameter τ_α_ obtains its justification from analogies in signal processing and in statistics in contrast to the WLS difference S_α_, whose minimality is necessary for estimating the parameter vector Θ. The misclassification rate D_α_ is relying on a priori expert knowledge.

Quality measures should quantify firstly the suitability of the input data (the separability of fat from muscle in a CT-image), secondly the efficacy of the segmentation (WLS fitting of the model X(Θ) to the histogram X and computation of the threshold t_FM_) and at last the correctness of the result (the anatomical correctness of FM segmentation). Our measures τ_α_, S_α_ and D_α_ cover all three aspects: τ_α_ measures the shape of the X(Y) to quantify the CT-image Y’s suitability for CFES application, S_α_ judges the final ‘distance’ between X and X(Θ) only, while D_α_ evaluates W*(Y) without distinction of genesis.

In^4^ we gave a synopsis of tissue segmentation studies which we review in Table 2 for their quality measures.

**Table 2:** Studies in literature about CT fat segmentation: quality measures.

Author, year, citation	Quality measure
Otsu^8^, 1979	Variance analysis inspired, threshold k optimization of η(k), η(k) : = V_Interclass_/V_Total of levels_ non-parametric
Mitsopoulos^9^, 1998	Comparison to sliced cadavers, correlation analysis
Rogalla^10^, 1998	Comparison to phantom, standard error
Yoshizumi^11^, 1999	Comparison to cadavers, planimetry, correlation analysis
Glasbey^12^, 2002	Comparison to simulated CT images, RMSE, bias, std.deviation
Dobrowolsky^13^, 2004; Romvary^14^, 2006	Comparison to butchered pigs, fat-muscle ratio, linear regression
Pednekar^15^, 2005	Comparison to manual segmented images, true negative/positive pixels
Johansen^16^, 2007	Comparison to estimation on carcasses, linear regression
Kongsro^17^, 2008	Comparison to measurement results of manual dissections, linear regression
McEvoy^18^, 2008	Std. dev. of local maximum estimator in grey-scale value histogram
Oshima^19^, 2008	No measure published
McEvoy^20^, 2009	No measure published
Checchini^21^, 2011	Correlation analysis estimated fat volume vs. BMI
Kanaly^22^, 2011	Comparison to informed observers results
Subramaniam^23^, 2012	Lung tissue inhomogeneity measurement method, validated by distinguishability of end-inspiration- and end-exspiration heterogeneity
Kim^24^, 2013	Comparison to dual-energy X-ray absorptiometry results, correlation analysis
Kullberg^25^, 2017	Comparison to manual CT-segmentation results, correlation analysis

Our choice of τ_α_, S_α_ and D_α_ may be partly explained since we were not able to determine a gold standard as e.g. manual dissection. S_α_ as the minimized quantity of quasi-segmentation seems to be a natural choice. As we knew the single material grey-scale value pdf to be Gaußian, we did not want to use non-parametric fitting methods as e.g.^8^. Usage of τ_α_ was inspired by the t-test statistic, used for deciding whether two Gaußians have different expectation values. Moreover there are similarities of τ_α_ to the signal theoretic quantity of signal to noise ratio (SNR), quantifying the separability of two signals in frequency domain. Finally the misclassification rate D_α_ seemed to be a canonical choice for a measure of the pure CFES-output quality.

In the slice of Fig. 2d–f the d_α_ implies good quality, whereas S_α_ indicates low quality, therefore we will investigated in more detail the interrelations between the three quality measures. Yet for α-calibration there is no better parameter than D_α_ or F_α_ since the usage of S_α_ as a quality measure for fat quantification runs into problems we will discuss below.

As CT numbers generally depend on temperature (see^26^), the question arises whether the results presented are robust against sample temperature changes. The results in^26^, using conventional polychromatic 120kVp CT (CPI) (as well as virtual monoenergetic images by dual energy measurements (VMI) with 40kVp, 50 kVp, 60 kVp, 70 kVp, 100 kVp, 120 kVp, 140 kVp), seem to give evidence to the hypothesis of robustness: The temperature-changes in an interval of [10 °C, 60 °C] led to changes of [− 40 HU, − 80 HU] in the grey-level values for fat-substitute and of [50 HU, 60 HU] for muscle-substitute in CPI (see Fig. 5 in^26^) which lie in the range of inter-personal grey-level value variations of our study. Furthermore^26^ indicates that there is no influence of the convolution kernel chosen on the temperature dependence of the grey-level values (see Fig. 5 in^26^) for CPI. The paper^26^ implies possibilities for further research on quasi-segmentation e.g. by using VMI and/or temperature control for improving CFES-results.

## Relations between the three quality measures

In Fig. 2a the sample’s location in the triangle tells us that high values of the μ_Yr_(τ_α_*) seem to force low values on D_α_ (see (8A)), whereas high values of D_α_ constrain μ_Yr_(τ_α_*) to low values (see (8B)). Nevertheless, there are abdominae with low values of D_α_ and of μ_Yr_(τ_α_*) (see (8C)):

8A $$\mu_{{\underline {Y} r}} \left( {\tau_{\alpha }^{*} } \right) \uparrow = > D_{\alpha } \downarrow$$

8B $$D_{\alpha } \uparrow = > \mu_{{\underline {Y} r}} \left( {\tau_{\alpha }^{*} } \right) \downarrow$$

8C $$\exists : D_{\alpha } \downarrow \wedge \mu_{{\underline {Y} r}} \left( {\tau_{\alpha }^{*} } \right) \downarrow$$

While (8A) and (8B) match our intuition (S) about relations of the quality measures, the last vertex of the triangle [(8C): low μ_Yr_(τ_α_*) and low D_α_] seems to contradict them grossly at first sight. Note however, that the minimum value of μ_Yr_(τ_α_*) for D_α_ = 0% is μ_Yr_(τ_α_*) ≈ 200. A ‘linear approximation’ to the upper bound of the relation is for α = 1:

9 $$0 \le D_{\alpha } \le A \cdot \mu_{{\underline {Y} r}} \left( {\tau_{\alpha }^{*} } \right) + B$$

where the line A μ_Yr_(τ_α_*) + B is estimated by connecting the two data-points (x_0_, y_0_) = (103.0 , 21.35%) for scan LFD_19 and (x_1_, y_1_) = (654.0, 0.0%) for scan LFD_23 by:

10 $$A^{*} = \frac{{y_{1} - y_{0} }}{{x_{1} - x_{0} }} = \frac{21.35\% - 0.0\% }{{103.0 - 654.0}} = - 0.03875\%$$

11 $$B^{*} = y_{1} - A^{*} \cdot x_{1} = 0.0 + 0.03875 \cdot 654.0 = 25.3425\%$$

Figure 2d–f displays slice 119 of scan LFD_5 as an example of a slice Y_r,l_ with low d_α_ and with low τ_α_* as well. This suggests the hypothesis of the threshold t_FM_ to be robust against fluctuations of τ_α_.

Figure 2b is a scatterplot of μ_Yr_(ln S_α_) vs. D_α_ for α = 1. As in Fig. 2a we see a relation, not a functional graph but a ‘formed cloud’ filling one corner of the first quadrant. In analogy to Fig. 2a we see on Fig. 2b high values of μ_Yr_(ln S_α_) seem to force low values of D_α_ [see (12A)], contradicting intuition (S), whereas high values of D_α_ constrain low values of μ_Yr_(ln S_α_) [see (12B)] contradicting (S) as well. The third corner of the triangle meets intuition (S): low μ_Yr_(ln S_α_) coincides with low D_α_ [see (12C)].

12A $$\mu_{{\underline {Y} r}} \left( {\ln S_{\alpha } } \right) \uparrow = > D_{\alpha } \downarrow$$

12B $$D_{\alpha } \uparrow = > \mu_{{\underline {Y} r}} \left( {\ln S_{\alpha } } \right) \downarrow$$

12C $$\exists :\mu_{{\underline {Y} r}} \left( {\ln S_{\alpha } } \right) \downarrow \wedge D_{\alpha } \downarrow$$

The upper boundary approximation line of the cloud is:

13 $$0 \le D_{\alpha } \le C \cdot \mu_{{\underline {Y} r}} \left( {ln S_{\alpha } } \right) + G$$

Estimating the line C μ_Yr_(ln S_α_) + G by connecting the data-points (x_0_, y_0_) = (21.62 , 21.35%) (scan LFD_19) and (x_1_, y_1_) = (23.88, 5.6%) (scan LFD_27) gives:

14 $$C^{*} = \frac{{y_{1} - y_{0} }}{{x_{1} - x_{0} }} = \frac{5.6\% - 21.35\% }{{23.88 - 21.62}} = - 6.97\%$$

15 $$G^{*} = y_{1} - C^{*} \cdot x_{1} = 5.6\% + 6.97\% \cdot 23.88 = 172.0\%$$

Figure 2c is the scatterplot (α = 1) of μ_Yr_(ln S_α_) vs. μ_Yr_(τ_α_*). The distinct linear relationship with positive slope was significant in by linear regression analysis, which is a contradiction (16) to (S):

16 $$\mu_{{\underline {Y} r}} \left( {\tau_{\alpha }^{*} } \right) \uparrow = > \mu_{{\underline {Y} r}} \left( {\ln S_{\alpha } } \right) \uparrow$$

The deviations (8C), (12A), (12B), (16) from (S) were investigated, using the simulated CT images (Y_A,r_)_r=1,…,R_ and (Y_B,r_)_r=1,…,R_ (see Fig. 3a, b).

Figure 3c shows a scatterplot of τ_α_^+^ vs. τ_α_* with ranges [0, 6000] on the X- and on the Y-axis for sample Y_A,r_. The matching of τ_α_^+^ and τ_α_* is almost perfect here, becoming slightly weaker in the region near (0,0). Zooming in on a range of [0, 400] for τ_α_^+^ we notice strong deviations of the estimator values τ_α_* in Fig. 3d. Note that the range of our sample Ψ’s mean separabilities μ_Yr_(τ_α_*) is [0, 600]: The correlation of τ_α_^+^ and τ_α_* of our sample Ψ would have to be estimated from the zoomed in representation in Fig. 3d rather than from the diagram in Fig. 3c. The correlation of τ_α_^+^ and τ_α_* refutes the objection to the positive correlation μ_Yr_(τ*_α_) − μ_Yr_(lnS_α_) in Fig. 2c: “Maybe *there is a negative* correlation in the analogous μ_Yr_(τ^+^_α_) − μ_Yr_(lnS_α_) diagram, which cannot be detected as τ^+^_α_ is not accessible for our real-world-sample Ψ, but the μ_Yr_(τ*_α_) − μ_Yr_(lnS_α_) diagram *shows a positive correlation* caused by a flaw of CFES generating a locally negative correlation between τ_α_^+^ and τ_α_*?” Despite the noise in Fig. 2c there is a distinctly positive relation, so the objection can be rejected.

The relations between τ_α_* and ln S_α_ are demonstrated in Fig. 4a as scatterplot of τ_α_* vs. ln S_α_ with α = 1 for the images Y_A,r_ (square marks). The *partial graph* over τ_α_*-range [1150, 3000] corresponds with (S): Rising τ_α_* is correlated with falling ln S_α_ and vice versa:

17 $$\forall \tau_{\alpha }^{*} \in \left[ {1150, 3000} \right]:\left( {\tau_{\alpha }^{*} \uparrow < = > \ln S_{\alpha } \downarrow } \right)$$

The curve in the range of [1150, 3000] is a function graph without random noise.

In the *complementary* partial graph over τ_α_*-range [0, 1150] the curve is more noisy. Its tendency contradicts (S) since rising τ_α_* coincides with rising ln S_α_:

18 $$\forall \tau_{\alpha }^{*} \in \left[ {0, 1150} \right]:\left( {\tau_{\alpha }^{*} \uparrow < = > \ln S_{\alpha } \uparrow } \right)$$

The two τ_α_*-ranges are associated to two ranges of S = S_F_ = S_M_ in the synthetic sample A: ‘τ_α_* in [1150, 3000]’ corresponds to ‘S in [10 HU, 25 HU] whereas ‘τ_α_* in [0, 1150]’ is associated to S ≥ 25 HU. Though ΔE = E_M_ − E_F_ = E_F_^+^  = 50 HU –(-80 HU) = 130 HU, even S = 25 HU means only a moderate grey-scale value distributions densities f_M_ and f_F_ overlap.

The area of the sample Ψ’s scatterplot μ_Yr_(τ*_α_) vs. μ_Yr_(lnS_α_) in Fig. 2c can be realized as the dashed rectangle in the τ_α_* vs. ln S_α_—scatterplot Fig. 4a: This area lies on the left side of [0, 1150] with trend (17) and comprises a vast variation of the ln S_α_—values in the scatterplot of the synthetic samples A and B. The trend in Fig. 2c seems to be fairly linear despite varying fat-proportions z in the real-worlds CT scan sample Ψ. It does not depend on the fat-proportion z, since the umbrella-form with the fuzzy left end shows up in both plots of Fig. 4a. While the top of the umbrella at about τ_α_* = 25 sank from ca. ln S_α_ = 25 to ca. ln S_α_ = 23.7 for z rising from z = 0.3 to 0.5, the maximum-location on the τ_α_*-axis is nearly identical.

To see, how the overlap between z∙f_F_ and (1 − z)∙f_M_ of a slice is associated to its position on the umbrella, Fig. 4b shows the τ_α_* vs. ln S_α_ scatterplot of (Y_A,r_)_r=0,…,R_ again. Additionally, we present five of the grey-scale value histograms X(Y), X(Θ), X_F_(Θ), X_M_(Θ) associated to the scatterplots data-points for the parameter values S = S_F_ = S_M_ = 12 HU, 25 HU, 60 HU, 100 HU, 150 HU. The umbrella’s top lies at S = 25 HU, and it is obvious, that the *increasing overlap* of the partial histograms X_F_(Θ) and X_M_(Θ) increasingly deteriorates z-estimation quality when moving from the right to the left side of the umbrella. The z-estimation quality can be recognized roughly from the overlap area under X_F_(Θ) and X_M_(Θ).

The importance of the umbrella—phenomenon can be demonstrated by relating it to the relative error RE_z_ : =|z* − z^+^|/z^+^ of our target variable z. In Fig. 4c, a 3-D scatterplot of τ_α_* vs. ln S_α_ vs. RE_z_ for (Y_A,r_)_r=1,…,R_ (black bubbles) and (Y_A,r_)_r=1,…,R_ (white bubbles) with the additional constraints: 5 HU ≤ S ≤ 100 HU and RE_z_ < 0.2 is shown. It demonstrates the abrupt strong rising of the error RE_z_ in the target variable z* if the data points leave the right side of the ‘umbrella’ in Fig. 4a (which can be found projected on the bottom of the coordinate cube in Fig. 4c as well) and proceed to the left side.

As reason for the umbrella form of the τ_α_* vs. ln S_α_ scatterplot (Fig. 4a), for the fuzziness and for the rise of the relative error RE_z_ on the left side of the umbrella (in Fig. 4c), a sort of *pseudo-overfitting* of the model f(y,Θ) can be hypothesized. While the well-known effect of overfitting is associated to using a model with too many parameters, our self-coined term ‘pseudo-overfitting’ means usage of a model with the correct number of parameters (in our case: 5 parameters: E_F_, E_M_, S_F_, S_M_, z) if the model is a mixture of distributions of the same type (in our case: the normal distribution mixture f(g,Θ) = z f_F_(g,Θ) + (1 − z) f_M_(g,Θ) ) and with an empirical distribution geometrically similar to a (mixture of less) distribution(s) of the same type (in our case: X(Y) seems to consist of only one normal distribution like in Fig. 4b the grey value distribution for S = 150 HU). Here we have to take into account similar effects as those caused by classical overfitting: The fitting process uses the seemingly redundant parameters to model some of the apparently random deviations, which leads to virtually getting ‘better’ results in terms of S_α_ though the separability τ_α_ decreases (see Figs. 2c and 4a). Associated with smaller values of S_α_ and τ_α_ one notes a dramatic loss in the estimators z*’s exactness which was measured by the relative error RE_z_ of the z-estimator z* (see Fig. 4d).

## A principal constraint in fitting Gaußian mixtures

A large proportion of the problems reported in 4.1 and 4.2 seems to come from a principal difficulty in fitting Gaußian mixtures as we did for estimating the parameter vector Θ: The grey value histogram X(Y) of a CT slice Y containing fat- and muscle components only was assumed to be a mixture of the Gaußian components X_F_ and X_M_ for fat F and muscle M respectively. Figure 4d demonstrates an exponential growth of the z-estimator’s standard deviation S(z*) for linearly increasing standard deviation S = S_F_ = S_M_ of the F- and the of the M-component. This is made plausible by comparing the graph on the left side of Fig. 4 (lower right part) (z = 0.5, 20 HU ≤ S ≤ 90 HU) to the graph on the right side (z = 0.5, 20 HU ≤ S ≤ 130 HU): though the curve seems to be geometrically identical, the ordinates range has grown by a factor of order 20 while the domain was enlarged from [20 HU, 90 HU] to [20 HU, 130 HU] only. This effect does not depend on the actual value of z: The shown graphs with a true parameter value of z = 0.3 are virtually indistinguishable from analogous ones–which therefore are not shown here—with true parameter value z = 0.5. The computation results presented in Fig. 4d strikingly demonstrate the fact that the exactness S(z*) of z-estimation is rapidly deteriorating with increasing overlap of the single-material—normal distributions in the grey-scale value histogram. Given a distance of ΔE = E_M_ − E_F_ = 50 HU − (− 80 HU) = 130 HU between the maxima in the histogram, a value of S = S_F_ = S_M_ ≥ 60 HU leads to an approximate z-estimator standard deviation S(z*) ≥ 0.1 while S ≥ 90 HU causes S(z*) ≥ 1.0. Note that z is a ratio from the interval [0, 1] which makes an estimator z* with S(z*) ≥ 1 nearly useless.

The difficulty in fitting Gaussian mixtures discussed above and particularly the swiftly growing standard deviation S(z*) of the z-estimator with rising standard deviation of S_F_ = S_M_ seems to be no consequence of our special fitting algorithm WLS chosen: Due to the high number of pixels in a CT slice it is possible to apply asymptotic propositions to the probability distribution of the estimator. Jennrich^28^ shows, that if the data’s (in our case the grey-value histogram X) probability distribution lies in the exponential family (the multinomial distribution of our case belongs to this family) the Fisher Scoring algorithm for iteratively finding the Maximum Likelihood Estimator (MLE) value is identical to the Gauss–Newton algorithm which iteratively detects the Non-linear Least Squares estimator’s (NLLS) value, which is detected by CFES as well. Hence the probability distribution of z* computed here via CFES (NLLS estimation respectively) is nearly identical to the distribution of the MLE of z which *asymptotically is the most effective estimator* (see^29,30^), at least in case α = 0.

Note further that the standard deviation S(z*)'s rapid rising with growing overlap between H_F_ and H_M_ are not reported in the fat segmentation literature (see^8–25^) except maybe hinted in^8^.

A practical consequence of this issue will be to choose CT parameter values minimizing the single-material distribution standard deviations S_F_ and S_M_.

## Supplementary Information

Supplementary Information.

## Data Availability

The datasets analysed during the current study are not publicly available since they consist of individual CT-scans. Each single slice is linked to a DICOM-file containing experiment parameter data and sensible case information. The DICOM-files are essential for the computations. The bodies were confiscated by the local prosecution who directed the CT scans for investigations.

## Abbreviations

M_j_: Material with index j (1 ≤ j ≤ J)
Ξ: Index for materials of interest: Ξ = F for ‘fat’ and Ξ = M for ‘muscle’
G: {0, … ,N-1} set of the N possible grey-scale values in CT slice
G_0_: Interval in G containing E_0_ = 0 HU and approximately all grey-scale values n in G indicating materials F or M
Q: Number of pixels in CT slice
Q_Ξ_: Number of pixels of material Ξ in CT slice
Q: {1, … ,Q} : = {1, … , Q_F_, Q_F_ + 1, … ,Q_M_ + Q_F_} index set of all Q = Q_F_ + Q_M_ pixels in a slice. The values Q_Ξ_ vary from slice to slice
Q_Ξ_: {1, … ,Q_Ξ_} index set of Ξ-pixels in slice with number Q_Ξ_ of Ξ-pixels after suitable renumbering with Ξ = F, M. The sets Q_Ξ_ vary from slice to slice
Y: (y_1_, … , y_Q_) Single CT slice with grey-scale values y_q_ at the q = 1,…Q pixels in G
Y_Ξ_: (y_Ξ1_, … , y_ΞQ_) partial CT slice of Ξ-pixels in slice Y
Ψ: (Y_1_, … ,Y_R_) sample of CT scans Y_r_ of R bodies’ abdomina with r = 1, … , R
Y_r_: (Y_r__,1_, … , Y_r,L__(r)_) sequence of slices Y_r,l_ with l = 1, … , L(r) of abdominal scan Y_r_
Y_r,l_: (Y_r,l,1_, … , y_r,l,Q_) sequence of pixel grey-scale values y_r,l,q_ in G of slice Y_r,l_ with q = 1, … , Q
Y_Λ__,r_: Series (r = 1,…,R) of simulated CT slices: z = 0.5 < = > : Λ = A; z = 0.3 < = > : Λ = B
E_Ξ_: Expectation value of Ξ-pixel grey-scale value in one slice Y_r__,l_
S_Ξ_: Standard deviation of Ξ-pixel grey-scale value in one slice Y_r__,l_
z: Q_F_/Q ratio of fat pixels or fat ratio in one slice Y_r,l_
RE_z_: |z^+^ − z*|/z^+^ relative error of estimator z*
Θ: (E_F_, E_M_, S_F_, S_M_, z) parameter vector of interest
Φ: Φ(y,E,S) Normal distribution pdf evaluated at grey-scale value y in G with expectation value E and standard deviation S
f_Ξ_: f_Ξ_(y,Θ) = Φ(y,E_Ξ_,S_Ξ_) grey-scale value pdf on G of material Ξ evaluated at grey-scale value y
f: f(y,Θ) = z f_F_(y,Θ) + (1-z) f_M_(y,Θ) overall grey-scale value pdf on G evaluated at grey-scale value y
X: X(Y) = (x_0_(Y), … , x_N-1_(Y)) empirical grey-scale value histogram of slice Y
X_Ξ_: Partial histogram of material Ξ in slice with: X = X_F_ + X_M_
X(Θ): Histogram computed by X(Θ) : = Q f(G ,Θ)
τ: τ(Y) fat-muscle separability
S: S_α_(X(Y),X(Θ)) WLS distance between X(Y) and X(Θ) with weight exponent α
I_j_: Interval in G with: For all pixels indices q: Estimation of quasi-segmentation: Pixel q associated with material M_j_ <=> y_q_ in I_j_
a_j_: Constant grey value associated with material M_j_ for quasi-segmentation
W(Y): Quasi-segmentation result for slice Y: Pixel image of same format as Y with: w_q_ : = a_j_ <=> y_q_ in I_j_ <=> pixel in W(Y) with index q associated with material M_j_
d: d_α_(Y,W*(Y)) FM misclassification of slice Y (d = 0 correctly classified, d = 1 misclassified)
D: D_α_(Y,W*(Y)) mean FM misclassification over all slices Y of scan Y
F: F_α_(Ψ,W*(Ψ)) mean sample FM misclassification over all scans Y of sample Ψ
t_FM_: Threshold fat/muscle in G
v: CT slices’ voxel volume
ρ_Ξ_: Mass density of material Ξ
m_Ξ_: V ρ_Ξ_ Q_Ξ_ mass of material Ξ

## Code availability

The program code is available from the corresponding author on reasonable request.

## References

[1] Hubig, M., Muggenthaler, H., and Mall, G. Chapter 6.2: Finite element method in temperature-based death time estimation, In: *Estimation of the Time Since Death* (ed. Madea, B.) 114–133, 3rd edn. (CRC Press, Taylor & Francis Group, 2016).

[2] Schenkl S (2017). Automatic CT-based finite element model generation for temperature-based death time estimation: Feasibility study and sensitivity analysis. Int. J. Legal Med..

[3] Weiser M (2018). Uncertainty in temperature-based determination of time of death. Heat Mass Transf..

[4] Hubig M (2018). Fully automatic CT-histogram-based fat estimation in dead bodies. Int. J. Legal Med..

[5] Fullerton, G. D. Fundamentals of CT Tissue Characterization, In: *Medical Physics of CT and Ultrasound: Tissue Imaging and Characterization* (eds. Fullerton, G. D. & Zagzebski, J. A.) 125–162 (AAPM Medical Physics Monograph No. 6, American Institute of Physics, 1980).

[6] Buzug TM (2004). Einführung in die Computertomographie: Mathematisch-physikalische Grundlagen der Bildrekonstruktion.

[7] Welch BL (1947). The generalization of "Student's" problem when several different population variances are involved. Biometrika.

[8] Otsu N (1979). A threshold selection method from gray-level histograms. IEEE Trans. Syst. Man Cybernet. SMC.

[9] Mitsiopoulos N (1998). Cadaver validation of skeletal muscle measurement by magnetic resonance imaging and computerized tomography. J. Appl. Physiol..

[10] Rogalla P, Meiri N, Hoksch B, Boeing H, Hamm B (1998). Low-dose spiral computed tomography for measuring abdominal fat volume and distribution in a clinical setting. Eur. J. Clin. Nutr..

[11] Yoshizumi T (1999). Abdominal fat: Standardized technique for measurement at CT. Radiology.

[12] Glasbey CA, Robinson CD (2002). Estimators of tissue proportions from X-ray CT images. Biometrics.

[13] Dobrowolski A, Romvari R, Allen P, Branscheid W, Horn P (2004). Schlachtkörperwertbestimmung beim Schwein – Röntgen-Computertomographie als mögliche Referenzmethode. Fleischwirtschaft.

[14] Romvári R, Dobrowolski A, Repa I (2006). Development of a computed tomographic calibration method for the determination of lean meat content in pig carcasses. Acta Vet Hung.

[15] Pednekar, A., Bandekar, A. N., Kakadiaris, I. A., & Naghavi, M. Automatic segmentation of abdominal fat from CT data, in *Proceedings of the Seventh IEEE Workshop on Applications of Computer Vision (WACV/MOTION’05), 2005 WACV/MOTIONS ’05*, vol 1, 308–315 (2005).

[16] Johansen J, Egelandsdal B, Roe M, Kvaal K, Aastveit AH (2007). Calibration models for lamb carcass composition analysis using computerized tomography (CT) imaging. Chemom. Intell. Lab. Syst..

[17] Kongsro J, Røe M, Aastveit AH, Kvaal K, Egelandsdal B (2008). Virtual dissection of lamb carcasses using computer tomography (CT) and its correlation to manual dissection. J. Food Eng..

[18] McEvoy FJ, Madsen MT, Strathe AB, Svalastoga E (2008). Hounsfield Unit dynamics of adipose tissue and non-adipose soft tissues in growing pigs. Res. Vet. Sci..

[19] Ohshima S (2008). Development of an automated 3D segmentation program for volume quantification of body fat distribution using CT. Jpn. J. Radiol. Technol..

[20] McEvoy FJ, Madsen MT, Nielsen MB, Svalastoga E (2009). Computer tomographic investigation of subcutaneous adipose tissue as an indicator of body composition. Acta Vet. Scand..

[21] Cecchini S, Cavazzini E, Marchesi F, Sarli L, Roncoroni L (2011). Computed tomography volumetric fat parameters versus body mass index for predicting short-term outcomes of colon surgery. World J. Surg..

[22] Kanaly CW (2011). A novel method for volumetric MRI response assessment of enhancing brain tumors. PLoS ONE.

[23] Subramaniam, K., Hoffman, E. A., & Tawhai, M. H. Engineering in Medicine and Biology Society (EMBC). In *2012 Annual International Conference of the IEEE San Diego, California USA, 28 August–1 September*, 4072–4089. 10.1109/EMBC.2012.6345869 (2012).

[24] Kim YJ (2013). Body fat assessment method using CT images with separation mask algorithm. J. Digit. Imaging.

[25] Kullberg J (2017). Automated analysis of liver fat, muscle and adipose tissue distribution from CT suitable for large-scale studies. Sci. Rep..

[26] Heinrich A, Schenkl S, Buckreus D, Güttler FV, Teichgräber UK (2021). CT-based thermometry with virtual monoenergetic images by dual-energy of fat, muscle and bone using FBP, iterative and deep learning–based reconstruction. Eur. Radiol..

[27] Schenkl, S. *Quantifizierung des menschlichen Fettgewebes für die temperaturgestützte Todeszeitschätzung mit der Finite-Elemente-Methode.* (Dissertation, University Hospital Jena – Friedrich-Schiller-University Jena, Germany, 2019)

[28] Jennrich RI, Moore RH (1975). Maximum likelihood estimation by means of nonlinear least squares. J. R. Stat. Soc. B.

[29] Papoulis A (1990). Probability & Statistics.

[30] Lehmann EL (1991). Theory of Point Estimation.

