# Supplementary material for: Quality measures for fully automatic CT histogram-based fat estimation on a corpse sample
Source: Sci Rep. 2022 Nov 23;12:20147. doi: 10.1038/s41598-022-24358-4 (PMC9684132; doi:10.1038/s41598-022-24358-4)
Supplement: Supplementary file 1 — Supplementary Information. [file 41598_2022_24358_MOESM1_ESM.pdf]

Supplementary Appendix

1. The normal equations of the WLS-problem

The estimator Θ* of the true parameter value Θ⁺ of parameter $\Theta := (\Theta_1, \ldots, \Theta_U)$ can be understood as defined by the formula:

$$\Theta^* := \operatorname*{argmin}_{\Theta} S_\alpha\big(X(Y), X(\Theta)\big) \tag{S1.1}$$

Determining Θ* via (A1) is called the *WLS problem*. Using the usual necessary condition for the minimum one gets:

$$\forall u = 1, \ldots, U: \frac{\partial S_\alpha\big(X(Y), X(\Theta)\big)}{\partial \Theta_u} = 0 \tag{S1.2}$$

This leads to the U *gradient equations*:

$$\forall u = 1, \ldots, U: \quad \frac{\partial S_\alpha\big(X(Y), X(\Theta)\big)}{\partial \Theta_u} = -\sum_{g \in G} \frac{\partial x_g(\Theta)}{\partial \Theta_u} \cdot \Big(x_g(Y) - x_g(\Theta)\Big) \cdot x_g(Y)^\alpha = 0 \tag{S1.3}$$

Let Θ be a parameter vector fulfilling (S1.3) and let $\Delta\Theta := (\Delta\Theta_1, \ldots, \Delta\Theta_U)$ be a parameter vector of small deviations from the solution Θ. A Taylor approximation of order 1 for the function $x_g(\Theta)$ leads to:

$$\forall g \in G: \; x_g(\Theta + \Delta\Theta) \approx x_g(\Theta) + \nabla x_g(\Theta) \cdot \Delta\Theta = x_g(\Theta) + \sum_{u=1}^{U} \frac{\partial x_g(\Theta)}{\partial \Theta_u} \cdot \Delta\Theta_u \tag{S1.4}$$

With the following definition of the Jacobi-matrix J(Θ) of the grey value histogram $X(\Theta) := (x_1(\Theta), \ldots, x_U(\Theta))$:

$$J(\Theta) := \begin{bmatrix} \frac{\partial x_1(\Theta)}{\partial \Theta_1} & \cdots & \frac{\partial x_1(\Theta)}{\partial \Theta_U} \\ \vdots & \cdots & \vdots \\ \frac{\partial x_N(\Theta)}{\partial \Theta_1} & \cdots & \frac{\partial x_N(\Theta)}{\partial \Theta_U} \end{bmatrix} \tag{S1.5}$$

equation (S1.4) can be rewritten:

$$X(\Theta + \Delta\Theta) \approx X(\Theta) + J(\Theta) \cdot \Delta\Theta \tag{S1.6}$$

which leads with $\Delta X(\Theta) := X(\Theta+\Delta\Theta) - X(\Theta)$ to:

$$\Delta X(\Theta) = X(\Theta + \Delta\Theta) - X(\Theta) \approx J(\Theta) \cdot \Delta\Theta \tag{S1.7}$$

and with the weight matrix $A := (\delta_{g,h} \cdot x_g(Y)^\alpha)_{g,h=1,\ldots,N}$ equation system (S1.3) becomes the *normal equations system* of the WLS-problem stated in (A1):

$$J(\Theta)^t \cdot A \cdot (\Delta X(\Theta) - J(\Theta) \cdot \Delta\Theta) = 0 \tag{S1.8}$$

Solving for ΔΘ finally yields:

$$\Delta\Theta = [J(\Theta)^t \cdot A \cdot J(\Theta)]^{-1} \cdot J(\Theta)^t \cdot A \cdot \Delta X(\Theta) \tag{S1.9}$$

2. The Jacobi matrix J(Θ) of the WLS-problem

As the expression for the g-th grey value $x_g(\Theta)$'s theoretical frequency may be written as the following konvex-combination:

$$x_g(\Theta) = Q \cdot z \cdot f(g, E_F, S_F) + Q \cdot (1-z) \cdot f(g, E_M, S_M) \tag{S2.1}$$

we yield for the partial derivations of $x_g(\Theta)$:

$$\frac{\partial x_g(\Theta)}{\partial z} = Q \cdot \left(f(g, E_F, S_F) - f(g, E_M, S_M)\right) \tag{S2.2}$$

$$\frac{\partial x_g(\Theta)}{\partial E_F} = Q \cdot z \cdot \frac{\partial f(g, E_F, S_F)}{\partial E_F} \tag{S2.3}$$

$$\frac{\partial x_g(\Theta)}{\partial E_M} = Q \cdot (1-z) \cdot \frac{\partial f(g, E_M, S_M)}{\partial E_M} \tag{S2.4}$$

$$\frac{\partial x_g(\Theta)}{\partial S_F} = Q \cdot z \cdot \frac{\partial f(g, E_F, S_F)}{\partial S_F} \tag{S2.5}$$

$$\frac{\partial x_g(\Theta)}{\partial S_M} = Q \cdot (1-z) \cdot \frac{\partial f(g, E_M, S_M)}{\partial S_M} \tag{S2.6}$$

With the following form of the normal density:

$$f(g, E, S) := \frac{1}{\sqrt{2 \cdot \pi} \cdot S} \cdot exp\left(-\frac{(g-E)^2}{2 \cdot S^2}\right) \tag{S2.7}$$

the derivations with respect to the parameters E and S can be computed:

$$\frac{\partial f(g, E, S)}{\partial E} := \frac{1}{\sqrt{2 \cdot \pi} \cdot S} \cdot exp\left(-\frac{(g-E)^2}{2 \cdot S^2}\right) \cdot \frac{g-E}{S^2} = f(g, E, S) \cdot \frac{g-E}{S^2} \tag{S2.8}$$

$$\frac{\partial f(g, E, S)}{\partial S} := \frac{1}{\sqrt{2 \cdot \pi} \cdot S} \cdot exp\left(-\frac{(g-E)^2}{2 \cdot S^2}\right) \cdot \left(\frac{(g-E)^2}{S^3} - \frac{1}{S}\right) = f(g, E, S) \cdot \left(\frac{(g-E)^2}{S^2} - 1\right) \cdot \frac{1}{S} \tag{S2.9}$$

The derivations (S2.3) – (S2.6) can therefore be computed explicitly:

$$\frac{\partial x_g(\Theta)}{\partial E_F} = Q \cdot z \cdot f(g, E_F, S_F) \cdot \frac{g-E_F}{{S_F}^2} \tag{S2.10}$$

$$\frac{\partial x_g(\Theta)}{\partial E_M} = Q \cdot (1-z) \cdot f(g, E_M, S_M) \cdot \frac{g-E_M}{{S_M}^2} \tag{S2.11}$$

$$\frac{\partial x_g(\Theta)}{\partial S_F} = Q \cdot z \cdot f(g, E_F, S_F) \cdot \left(\frac{(g-E_F)^2}{{S_F}^2} - 1\right) \cdot \frac{1}{S_F} \tag{S2.12}$$

$$\frac{\partial x_g(\Theta)}{\partial S_M} = Q \cdot (1-z) \cdot f(g, E_M, S_M) \cdot \left(\frac{(g-E_M)^2}{{S_M}^2} - 1\right) \cdot \frac{1}{S_M} \tag{S2.13}$$

The Jacobi-matrix J(Θ) from (A.5) is now determined via (S2.2), (S2.10), (S2.11), (S2.12), (S2.13) and (S2.7).

3. The covariance matrix Cov(Θ)

In our stochastic model approach we assume X to be the grey value histogram which leads via (A.1) to an estimated parameter vector Θ and ΔX to be a grey value difference histogram which is caused by the 'real world noise' generating the histogram X instead of the true histogram X^+:

$$\Delta X \coloneqq X - X^+ \tag{S3.1}$$

As we further assume the noisy histogram X to correspond to an estimated parameter vector Θ as well as the true histogram X^+ to a parameter vector Θ^+, we can define the parameter vector difference ΔΘ:

$$\Delta\Theta \coloneqq \Theta - \Theta^+ \tag{S3.2}$$

Since (A.9) this implies for the covariance matrices Cov(Θ) = Cov(ΔΘ) and Cov(X) = Cov(ΔX) the following connection:

$$Cov(\Theta) = [J(\Theta)^t \cdot A \cdot J(\Theta)]^{-1} \cdot J(\Theta)^t \cdot A \cdot Cov(X) \cdot A \cdot J(\Theta) \cdot [J(\Theta) \cdot A \cdot J(\Theta)^t]^{-1} \tag{S3.3}$$

Formula (S3.3) is derived by simply writing the definition of Cov(ΔΘ) and inserting (A.11).

4. The covariance matrix Cov(X)

Let Y be a specific CT-image slice of an abdomen in our sample, X be the actual empirical grey value histogram and let Θ = (E_F, S_F, E_M, S_M, z) be the value of X's parameter vector. Computing the covariance matrix Cov(X) of the histogram X starts from the theoretical histogram X(Θ), given the true value Θ of the complete parameter vector Θ. We hypothesize the actual histogram X of the image Y generated by Q independent drawings of balls b_1, … , b_Q from an urn U with replacement. Each ball b in the urn is labelled by a grey-scale value g(b) from G = {0, … , N-1}: The ratio of balls b in U labelled with an arbitrary fixed grey-scale value g = g(b) is assumed to be f(g,Θ). After Q drawings the empirical histogram X shall be computed by defining:

$$\forall g \in G\colon\ X_g \coloneqq |\{b_q \,|\, q = 1, \dots, Q \ \wedge\ g(b_q) = g\}| \tag{S4.1}$$

It is evident from its hypothetical genesis that the empirical histogram X = $(x_n)_{n\in\underline{G}}$ follows the multinomial distribution P_X:

$$P_X \coloneqq \frac{Q!}{\prod_{n\in\underline{G}} x_n!} \cdot \prod_{n\in\underline{G}} f(n,\Theta)^{x_n} \tag{S4.2}$$

with the well-known covariance matrix Cov(X):

$$Cov(X) = (C_{k,n})_{k,n=0,\dots,N-1} \coloneqq \begin{Bmatrix} Q \cdot f(k,\Theta) \cdot (1 - f(k,\Theta)) & for & k = n \\ -Q \cdot f(k,\Theta) \cdot f(n,\Theta) & & k \neq n \end{Bmatrix} \tag{S4.3}$$
